# CPP‐E1A fusion peptides inhibit CtBP‐mediated transcriptional repression

**DOI:** 10.1002/1878-0261.12330

**Published:** 2018-06-23

**Authors:** Melanie A. Blevins, Caiguo Zhang, Lingdi Zhang, Hong Li, Xueni Li, David A. Norris, Mingxia Huang, Rui Zhao

**Affiliations:** ^1^ Department of Biochemistry and Molecular Genetics University of Colorado Denver Anschutz Medical Campus Aurora CO USA; ^2^ Department of Dermatology University of Colorado Denver Anschutz Medical Campus Aurora CO USA

**Keywords:** cell‐penetrating peptides, CtBP, CtBP inhibitors, transcriptional corepressors

## Abstract

The carboxyl‐terminal binding proteins (CtBP) are transcriptional corepressors that regulate the expression of multiple epithelial‐specific and pro‐apoptotic genes. Overexpression of CtBP occurs in many human cancers where they promote the epithelial‐to‐mesenchymal transition, stem cell‐like features, and cell survival, while knockdown of CtBP in tumor cells results in p53‐independent apoptosis. CtBPs are recruited to their target genes by binding to a conserved PXDLS peptide motif present in multiple DNA‐binding transcription factors. Disrupting the interaction between CtBP and its transcription factor partners may be a means of altering CtBP‐mediated transcriptional repression and a potential approach for cancer therapies. However, small molecules targeting protein–protein interactions have traditionally been difficult to identify. In this study, we took advantage of the fact that CtBP binds to a conserved peptide motif to explore the feasibility of using peptides containing the PXDLS motif fused to cell‐penetrating peptides (CPP) to inhibit CtBP function. We demonstrate that these peptides disrupt the ability of CtBP to interact with its protein partner, E1A, in an AlphaScreen assay. Moreover, these peptides can enter both lung carcinoma and melanoma cells, disrupt the interaction between CtBP and a transcription factor partner, and inhibit CtBP‐mediated transcriptional repression. Finally, the constitutive expression of one such peptide, Pep1‐E1A‐WT, in a melanoma cell line reverses CtBP‐mediated oncogenic phenotypes including proliferation, migration, and sphere formation and limits tumor growth *in vivo*. Together, our results suggest that CPP‐fused PXDLS‐containing peptides can potentially be developed into a research tool or therapeutic agent targeting CtBP‐mediated transcriptional events in various biological pathways.

**Abbreviations**
CPP: cell‐penetrating peptides
CtBP: C‐terminal binding protein
EMT: epithelial‐to‐mesenchymal transition
NADH: nicotinamide adenine dinucleotide
PLA: proximity ligation assay
WT: wild‐type

## 1 Introduction

C‐terminal binding proteins (CtBP) are transcriptional corepressors that regulate the expression of numerous genes critical for both developmental and oncogenic processes. CtBP was originally identified as a protein partner of the adenovirus protein, E1A, binding to a short peptide motif, PXDLS, located in the C‐terminal region of E1A (Boyd *et al*., 1993; Schaeper *et al*., 1995). Later, it was determined that CtBP binds a variety of protein partners through this conserved motif, including DNA‐binding transcription factors that play broad roles in tissue morphogenesis (e.g., Smad6, Lin *et al*., 2003; SOX6, Murakami *et al*., 2001; EVI1, Izutsu *et al*., 2001; δEF1, Furusawa *et al*., 1999; and ZEB1/2, Gheldof *et al*., 2012; Postigo and Dean, 1999) as well as chromatin‐remodeling enzymes and protein complexes that recruit epigenetic regulators (e.g., HDAC4/5/7, Zhang *et al*., 2001; MITR, Zhang *et al*., 2001; WIZ, Ueda *et al*., 2006; and p300 among others, Chinnadurai, 2007, 2009; Kim *et al*., 2005). The mammalian genomes contain two CtBP genes, *CtBP1* and *CtBP2,* which display distinct and overlapping roles in development. CtBP1/2 resembles a dehydrogenase structurally, although the physiological function of the dehydrogenase activity and its relationship to the transcriptional suppression activity of CtBP1/2 remain poorly defined. CtBP1/2 can bind the nicotinamide adenine dinucleotide (NADH/NAD+) cofactors, which promotes the homo‐ and heterodimerization of CtBP1/2 in a way that positions the PXDLS‐binding domains at opposites ends of the dimer. As a result, a possible mechanism for CtBP1/2 in regulating gene transcription lies in their ability to bridge various PXDLS‐containing DNA‐binding transcriptional factors and chromatin‐modifying proteins to form a large transcriptional repression complex (Chinnadurai, 2007). Fittingly, mutations within either the PXDLS‐binding sequence or CtBP's binding interface can significantly reduce CtBP's ability to interact with its protein partners and subsequent gene repression (Molloy *et al*., 1998, 2007; Nardini *et al*., 2003).

The ability of CtBP1/2 to alter various gene networks that regulate cellular differentiation, proliferation, and survival suggests that CtBP1/2 overexpression in adult tissue could promote both tumorigenesis and tumor progression. This idea is strengthened by several studies that have found a correlation between CtBP1/2 overexpression and cancer progression in multiple tumor types, including blood (Senyuk *et al*., 2002), skin (Deng *et al*., 2013), breast (Birts *et al*., 2010; Deng *et al*., 2012; Di *et al*., 2013), prostate (Moiola *et al*., 2014; Wang *et al*., 2012), ovarian (May *et al*., 2013; Zhang *et al*., 2017), and colon cancers (Pena *et al*., 2005, 2006). In addition, cancer cells typically have elevated NADH levels due to both hypoxia and pseudo‐hypoxia (NADH production when oxygen is not limited) (Sattler *et al*., 2007; Yeung *et al*., 2008; Zhang *et al*., 2002, 2006). As NADH binds to CtBP1 with high affinity (*K*_d_ = 100 nm) and causes a conformation change to favor its binding to the transcriptional repressors (Zhang *et al*., 2002), high NADH levels under hypoxic conditions associated with solid tumors can stimulate CtBP1 activity (Zhang *et al*., 2006).

A genomewide CtBP ChIP‐Seq study in breast cancer cells has identified over 1800 potential CtBP target genes and revealed that CtBP drives epithelial‐to‐mesenchymal transition (EMT), stem cell pathways, and genome instability (Di *et al*., 2013). This finding is significant given that the dysregulation of these cellular programs can promote tumorigenesis through increasing cellular proliferation, migration/invasion, and drug resistance, and suggests that inhibiting CtBP function may also inhibit tumorigenesis and metastasis. Supporting this notion, Ichikawa and colleagues found that the MCRIP1 protein, which contains the PXDLS motif, impedes CtBP‐mediated transcriptional repression through its ability to competitively inhibit the interaction between CtBP and its transcription factor partner, ZEB1 (Ichikawa *et al*., 2015). This MCRIP1‐CtBP association is disrupted by ERK‐mediated phosphorylation of MCRIP1, freeing CtBP to interact with ZEB1, thereby promoting transcriptional repression. Importantly, the ectopic expression of the unphosphorylatable MCRIP1(AA) mutant can inhibit EMT, suggesting that inhibiting the interaction between CtBP and its transcription factor partners could be an effective approach to reverse CtBP‐mediated oncogenic phenotypes. In addition, blocking CtBP efficiently triggers apoptosis of p53‐mutant/null cancer cells that would otherwise escape therapies that require p53‐mediated apoptosis (Paliwal *et al*., 2006; Zhang *et al*., 2005).

Attempts have been made to identify molecules targeting CtBP‐mediated transcriptional repression through multiple angles (Blevins *et al*., 2017). For example, phenylpyruvate analogs have been generated that indirectly inhibit CtBP‐mediated transcription suppression through inhibiting the enzymatic activity of CtBP, with limited cellular potency (IC50s ranging from 0.85 to 4 mm) (Hilbert *et al*., 2015; Korwar *et al*., 2016; Sumner *et al*., 2017). A second approach utilizes a cyclic peptide, CP61, which prevents the dimerization and function of CtBP in MCF7 cells at 50 μm (Birts *et al*., 2010). Given the extremely high attrition rate of drug discovery (Arrowsmith, 2012), it is difficult to predict whether these preliminary inhibitors can be developed into a drug eventually.

Short (14‐mer) E1A peptides have been shown to be capable of disrupting the CtBP‐E1A interaction with low micromolar *K*_d_ values in a competitive ELISA (Molloy *et al*., 1998). Therefore, E1A peptides may also reverse CtBP‐mediated oncogenic phenotypes *in vivo*. Generally, peptides like the 14‐mer E1A peptide cannot enter cells to elicit an effect. On the other hand, cell‐penetrating peptides (CPP) are typically short highly charged peptides (10–30 amino acids in length) that can penetrate the cell membrane and enter almost all cell types (Sebbage, 2009). CPPs can carry a number of conjugated molecular cargo into the cell, including DNA, RNA, peptides, and proteins (Sebbage, 2009). Therefore, a CPP conjugated to a peptide (such as the CtBP‐binding motif) can be a useful approach toward targeting protein–protein interactions in transcription factor complexes.

In this study, we evaluate the feasibility of targeting CtBP‐overexpressing tumors through a therapeutic peptide capable of disrupting the CtBP transcription factor complex, using the H1299 non‐small cell lung cancer cells and the A375 melanoma cells for *in vitro* experiments and a xenograft mouse melanoma model for *in vivo* experiments. CtBP1 has been shown to be overexpressed in melanoma and is important for melanoma development (Deng *et al*., 2013). Although CtBP overexpression has not been reported in lung cancers yet, previous studies by several groups (Madison and Lundblad, 2010; Paliwal *et al*., 2012; Wang *et al*., 2006) have established the lung cancer cell line H1299 as an *in vitro* model system for characterization of CtBP's function as a transcriptional corepressor of genes involved in cell cycle arrest (e.g., p21) and apoptosis (e.g., BAX). In particular, CtBP activity has been shown to be critical to cellular survival of H1299 (Wang *et al*., 2006). Moreover, H1299 cells lack p53, while the melanoma cell line A375 bears wild‐type (WT) p53. Therefore, these two cell lines serve complementary purposes for investigation of CtBP's role in both p53‐dependent and p53‐independent pathways that regulate cellular proliferation and survival.

We have designed and produced E1A peptides that contain the PXDLS motif and can disrupt the CtBP1–protein partner interaction *in vitro*. The addition of a CPP to the N terminus facilitated entry of the E1A peptide into cells where they were able to disrupt the CtBP complex and relieve CtBP‐mediated transcriptional repression. Additionally, the constitutive expression of these peptides in both human and mouse melanoma cell lines suppressed cell proliferation, migration, and stem cell‐like features associated with CtBP1 overexpression and decreased tumor growth in a xenograft growth assay using stably transfected mouse melanoma cells. These results open up the exciting possibility of using the CPP‐E1A peptide as a strategy by which to alter CtBP‐mediated transcriptional repression and tumorigenesis.

## 2 Materials and methods

### 2.1 Peptide expressions and purifications

Peptide‐coding sequences were subcloned into a modified pET15b vector (Kendrick *et al*., 2014), in which the sequence encoding the Protein G B1 domain (GB1 is a highly soluble protein domain that facilitates the expression of peptides in *Escherichia coli*, Cheng and Patel, 2004) was subcloned upstream of the 6xHis tag, followed by the CPP‐E1A peptide sequence, and a FLAG tag. To generate the mutant E1A peptide (E1A‐EL), a modified QuikChange site‐directed mutagenesis protocol (Liu and Naismith, 2008) was used to alter the amino acids LS to EL in the PXDLS sequence.

Each peptide fusion was expressed in BL21 (DE3) *E. coli* at 23 °C with 1 mm IPTG induction for 5 h. Cells were harvested and lysed by sonication in Buffer L (50 mm Tris [pH 8.0], 250 mm NaCl, 5% glycerol, and 1 mm DTT) including the protease inhibitors: 1 μg·mL^−1^ pepstatin A, 1 μm leupeptin, and 1 mm PMSF. Following centrifugation, the supernatant was loaded onto Ni^2+^‐NTA agarose resin (Invitrogen, Carlsbad, CA, USA) and eluted from the column using 400 mm imidazole. The peptides were then cleaved overnight at 16 °C with thrombin while dialyzing into Buffer L to remove the GB1‐6xHis tag. The peptides were dialyzed once more to reduce the imidazole concentration to below 1 mm before being reloaded onto the Ni^2+^ column to separate the GB1‐6xHis from the peptides. The peptides in the flow‐through were further purified by reversed‐phase high‐performance liquid chromatography (RP‐HPLC) on a Zorbax SB‐300 C8 column (250 × 9.4 mm inner diameter, 5 μm particle size) using an Agilent 1100 Series HPLC (Agilent, Santa Clara, CA, USA). Fraction purity was confirmed by electrospray mass spectrometry using Agilent 1100 Series LC‐MSD Trap (Agilent) before being pooled and lyophilized. The TAT alone peptide was synthesized by solid‐phase synthesis in the Protein and Peptide Core Facility (University of Colorado, Anschutz Medical Campus).

### 2.2 AlphaScreen assays

The AlphaScreen assay was carried out using 6xHis‐CtBP1 and GST‐E1A proteins following the manufacturer's protocol (PerkinElmer, Waltham, MA, USA) unless otherwise specified. GST‐E1A and 6xHis‐CtBP were purified as previously described (Blevins *et al*., 2015). To confirm inhibition by CPP‐E1A peptides, each peptide was added at varying concentrations (2 μm to 200 μm) to compete with GST‐E1A in the AlphaScreen assay containing 125 nM 6xHis‐CtBP1 and 125 nm GST‐E1A. The IC_50_ value of each peptide was calculated using the graphpad prism software (GraphPad Software, San Diego, CA, USA).

### 2.3 Cell culture

For experiments targeting the CtBP1 complex, the human H1299 non‐small cell lung carcinoma as well as the human A375 and mouse B16‐F0 melanoma cell lines were cultured in Dulbecco's modified Eagle's medium (DMEM; Corning, Tewksbury, MA, USA) containing 10% fetal bovine serum (FBS; Corning), 2 mm l‐glutamine (HyClone, Logan, UT, USA), and 1% penicillin/streptomycin (Corning) unless otherwise indicated. Both H1299 and A375 cell lines were authenticated using STR DNA fingerprinting and mycoplasma tested using the MycoAlert Mycoplasma Detection Kit (Lonza Group Ltd, Basel, Switzerland) on March 21, 2017. The B16‐F0 cell line was from ATCC (Manassas, VA, USA) (CRL‐6322).

To generate stably transfected A375 and B16‐F0 cell lines, the CPP‐E1A WT and CPP‐E1A‐EL peptide‐coding sequences were subcloned into the pCDNA3.1 vector between BamHI and XhoI sites. The PCR primers used for the amplification of both CPP‐E1A and its mutant from their parental vectors are F: 5′‐CGCGGATCCAAAGAAACCTGGTGGGAAACCTGG‐3′ and R: 5′‐CCGCTCGAGTTACTTGTCGTCATCGTCTTTGTA‐3. Cells were seeded at 1.0 × 10^5^ per well in a 24‐well plate in 500 μL of growth medium (DMEM with 10% FBS). pcDNA3.1, pcDNA3‐Pep1‐E1A‐WT, or pcDNA3‐Pep1‐E1A‐EL were transfected with Lipofectamine 2000 following the manufacture's guidelines (ThermoFisher Sci, Waltham, MA, USA). Cells were selected using medium containing G418 in the concentration range of 0.1–1.0 mg·mL^−1^. G418‐resistant cells were collected after 2 weeks of selection.

### 2.4 Luciferase reporter assays

The luciferase assays were carried out in H1299 and A375 cells as previously described (Blevins *et al*., 2015), with the following changes: Cells were treated with each peptide at varying concentrations for 8 h before being lysed with passive lysis buffer and analyzed for Firefly and *Renilla* luciferase activity.

### 2.5 Reverse transcription and quantitative PCR (RT‐qPCR)

Total RNA was isolated using TRIzol (Invitrogen) and reverse‐transcribed into cDNAs using the Random Primer Mix and ProtoScript II Reverse Transcriptase (New England Biolabs, Ipswitch, MA, USA). RT‐qPCR was performed using a SYBR Green I Master Mix (Roche, Indianapolis, IN, USA) and amplified using a LightCycler 480 (Roche). The relative RNA expression levels were determined by normalizing to 18S rRNA; values were calculated using the $\begin{array}{c} 2^{-\Delta C_{r}} \end{array}$ method (Livak and Schmittgen, 2001). The following primer sequences were used for the four target genes: 18S‐F: TGA CGG AAG GGC ACC ACC AG, 18S‐R: GCA CCA CCA CCC ACG GAA TC, BAX‐F: CCC CGA TTC ATC TAC CCT GCT G, BAX‐R: TTG AGC AAT TCC AGA GGC AGT GG, BRCA1‐F: TTC TGG CTT CTC CCT GCT CAC AC, BRCA1‐R: GGC AAC ATA CCA TCT TCA ACC TCT GC, E‐cadherin‐F: GCA GCC AAA GAC AGA GCG GAA C, and E‐cadherin‐R: ACC CAC CTC AAT CAT CCT CAG CA. Three independent experiments were performed, and samples were assayed in triplicate for each experiment. Data are presented as mean ± standard deviation from the averaged values of each experiment.

### 2.6 Cell fractionation and CPP‐E1A peptide subcellular localization

For cellular fractionation, H1299 cells were treated with 42 μg·mL^−1^ digitonin in NEH lysis buffer (20 mm HEPES [pH 7.4], 150 mm NaCl, 0.2 mm EDTA, 2 mm DTT, and 2 mm MgCl_2_) plus a protease inhibitor cocktail (Roche) for 10 min at 4 °C. The H1299 cell lysate was centrifugated at 15 000 ***g*** in a Beckman Coulter Microfuge 22R (Beckman Coulter, Brea, CA, USA) for 10 min at 4 °C to remove the cytosolic fraction. The remaining pellet was washed twice with chilled PBS before the addition of the nuclear lysis buffer (40 mm Tris/HCl [pH 7.4], 100 mm NaCl, 10% glycerol, 2 mm β‐ME, and 0.5% Triton X‐100) plus the protease inhibitor cocktail. The nuclear fraction was incubated at 4 °C for 1 h before the final centrifugation at 15 000 ***g*** for 20 min. The soluble nuclear fraction was then used for a western blot analysis to monitor peptide levels within the nuclear fraction using a FLAG antibody (LT0420; LifeTein LLC, Somerset, NJ, USA). The western blot was also probed with primary antibodies against α‐tubulin (sc‐8035; SantaCruz Biotechnology, Dallas, TX, USA) and HDAC1 (ab‐68436; Abcam, Cambridge, MA, USA) as cytoplasmic and nuclear controls, respectively, to confirm subcellular fractionation.

### 2.7 Immunofluorescence assay

H1299 or A375 cells were allowed to adhere to a cover slip overnight in DMEM complete media and grown until they reached approximately 75% confluency on the cover slip surface. After a 30 min treatment with 20 μm peptides, TAT (used as a negative control), TAT‐E1A‐WT, and Pep1‐E1A‐WT, cells were fixed for 10 min using 100% methanol, and permeabilized for 1 min in 100% acetone. Each sample was stained with an anti‐FLAG antibody and detected using an Alexa Fluor 555 anti‐mouse IgG (4409S; Cell Signaling Technology, Danvers, MA, USA). DAPI staining was used to visualize the nucleus.

### 2.8 Proximity ligation assay (PLA)

H1299 or A375 cells were fixed and incubated for 1 h with 20 μg·mL^−1^ of either anti‐FLAG and anti‐CtBP1, or 20 μg·mL^−1^ of anti‐ZEB1 and anti‐CtBP1 primary antibodies at room temperature (FLAG antibody, mouse monoclonal, F1804, Sigma‐Aldrich (St Louis, MO, USA); ZEB1 antibody, mouse monoclonal, 2A8A6, Novusbio; and CtBP1 antibody, rabbit polyclonal; 07‐306, EMD Millipore). The interaction between CtBP1 and either the FLAG‐tagged peptides or ZEB1 was monitored using the Duolink *In Situ* PLA according to the manufacturer's protocol. Briefly, after washing with Tris‐buffered saline, secondary antibodies conjugated with oligonucleotides (Duolink *In Situ* PLA Probe Anti‐Mouse Minus and Duolink *In Situ* PLA Probe Anti‐Rabbit Plus; Sigma‐Aldrich) were added and incubated in a preheated humidity chamber for 1 h at 37 °C. This step was followed by ligation, amplification, and detection (Duolink *In Situ* Detection Reagents; Sigma‐Aldrich). Nuclear staining solution was added to the slides for 10 min prior to imaging. Each experiment was replicated two times and a minimum of three pictures per condition (magnification: ×100) were taken using the Leica DM6 microscope and leica application suite x software (Leica Microsystems Inc., Buffalo Grove, IL, USA). The PLA signals and nuclei were processed and quantified using imagej (Abramoff *et al*., 2004) and cellprofiler (Kamentsky *et al*., 2011) softwares. PLA signals/field and mean number of PLA signals/nucleus were calculated for each case.

### 2.9 Proliferation assay

These experiments were performed with A375 and B16‐F0 cells stably transfected with pCDNA3, pCDNA3‐Pep1‐E1A‐WT and pCDNA3‐Pep1‐E1A‐EL. Cells were seeded at a low density of 3000 cells/well in 24‐well plates. After 0, 24, 48, 72, 96, and 120 h, cells were formalin‐fixed and stained with crystal violet to evaluate cellular proliferation as described (Feoktistova *et al*., 2016).

### 2.10 Migration assay

A375 and B16‐F0 cells were grown in RPMI1640 medium to ~ 80% confluency, and then treated with 5 μg·mL^−1^ of mitomycin C for 2 h. Cells were then scratched and washed with PBS, and fresh RPMI1640 was added (Li *et al*., 2004). Images were taken at 0 and 24 h.

### 2.11 Sphere formation assay

Stable cell lines were incubated for 24 h, then diluted and seeded into 96‐well plates at 100 cells/well. Cells were grown in DMEM‐F12 supplemented with 1% B27, 20 ng·mL^−1^ epidermal growth factor (EGF), 20 ng·mL^−1^ basic fibroblast growth factor (bFGF), and 4 μg·mL^−1^ heparin, then photographed after 9 days as described (Mukherjee *et al*., 2015).

### 2.12 *In vivo* tumor growth assay

Mice were housed at the Center for Comparative Medicine at the UCD Anschutz Medical Campus and cared for in accordance with the humane care and use of laboratory animals guide. This study was performed according to protocols reviewed and approved by the Institutional Animal Care and Use Committee at the UCD Anschutz Medical Campus. The mice used in this study were C57BL/6 males from the Jackson Laboratory, at an average age of 16–17 weeks and 30–35 g body weight. Mice were randomly divided into three groups (5 mice per group, 2 injection sites per mouse, *n* = 10). Mouse numbers were determined using Clara Sample Power Analysis. Ten injection sites in each group will give 90% power, with alpha at 0.05 to detect difference of 25% tumor burden with a standard deviation of 15%. B16‐F0 cells were injected subcutaneously in 100 μL of a 4 : 1 mixture of sterile DMEM: growth factor reduced Matrigel (354230; Corning) on the back flanks of C57BL/6 male mice. Mice were injected with 1 × 10^7^ B16‐F0 cells stably transfected with pcDNA3, pcDNA3‐Pep1‐E1A‐WT, or pcDNA3‐Pep1‐E1A‐EL. Tumor volume (length × (width^2^)/2) was monitored every other day by a caliper measurement. All mice were euthanized 21 days after injection or when they became moribund as described (Overwijk and Restifo, 2001).

### 2.13 Statistical analysis

All bar graphs were created with graphpad prism software. Data are presented as the mean ± SD. In all statistical analyses, two‐sided tests were applied. Statistical significance was determined by Student's *t*‐test and *P* values < 0.05 were considered as statistically significant.

## 3 Results

### 3.1 The design of CPP‐E1A fused peptides

**Figure 1:**
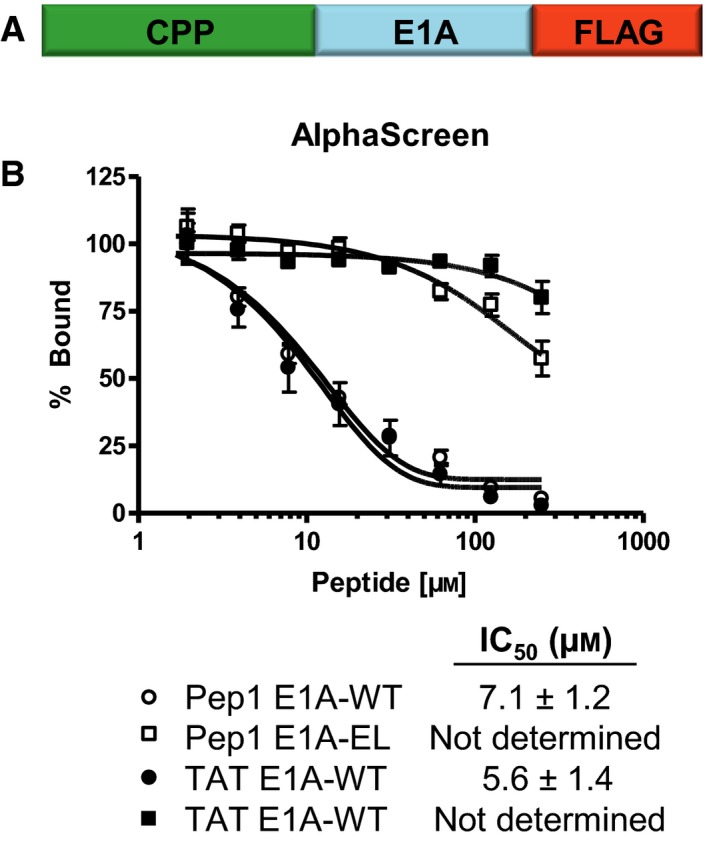
The CtBP1–E1A interaction is disrupted by CPP‐fused E1A peptides. (A) A schematic representation of the CPP‐fused E1A peptide design. (B) Competitive disruption of the CtBP1–E1A interaction by CPP‐fused E1A WT and mutant EL peptides as monitored by an AlphaScreen assay. Each data point is the representative result from three experiments performed in triplicate, and error bars represent the standard deviation for each concentration point.

Previous studies have shown that a 14‐mer E1A peptide containing the PXDLS motif and its derivatives are capable of disrupting the CtBP‐E1A interaction with *K*_D_ values ranging from 1.5 to 20.4 μm in competitive ELISAs (Molloy *et al*., 1998). Therefore, we designed a set of peptides around the PXDLS‐binding sequence found in the adenovirus 5 E1A oncoprotein (designated as E1A‐WT, Table 1 and Fig. 1A). Another series of peptides containing two point mutations in the CtBP‐binding motif (LS to EL, designated as E1A‐EL) were also constructed to serve as negative controls (Table 1). These two point mutations were shown to dramatically reduce the E1A peptide affinity by Molloy and colleagues (Molloy *et al*., 1998). For these peptides to enter cells, two CPPs, TAT and Pep1, were added to the N‐terminal end of the E1A peptides (Table 1 and Fig. 1A). TAT and Pep1 are two of the best characterized CPPs (Trabulo *et al*., 2010) and were chosen for our initial peptide design. A FLAG tag was also incorporated on the C‐terminal end of the peptides to facilitate detection of peptide internalization, stability, and localization by fluorescence microscopy and western blot analyses. Due to their relatively large size we chose to produce these peptides through bacterial expression and purification using Ni^2+^ affinity chromatography followed by reverse phase‐HPLC.

**Table 1:**
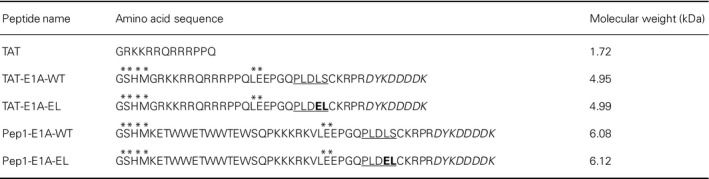
Sequences of peptides used in this study

* Indicates residues from cloning; bold residues are mutated residues; underlined residues indicate residues important for CtBP1 binding; italicized residues are the FLAG tag epitope.

### 3.2 CPP‐E1A‐WT peptides disrupt the CtBP1‐E1A interaction in the AlphaScreen assay

Previously, we have shown that the E1A peptide is capable of disrupting the CtBP1‐E1A interaction using both an AlphaScreen and an ELISA (Blevins *et al*., 2015). To determine if the addition of both an N‐terminal CPP and a C‐terminal FLAG tag would alter the ability of the E1A peptide to disrupt the CtBP1‐E1A interaction, we preformed an additional AlphaScreen assay. WT E1A fused to either TAT or Pep1 with the C‐terminal FLAG was able to disrupt the CtBP1‐E1A interaction with IC_50_ values of 5.6 ± 1.4 and 7.1 ± 1.2 μm, respectively, while the corresponding EL mutants showed only modest inhibition at the highest concentrations of 250 μm (Fig. 1B). These results demonstrate that the addition of a CPP and a FLAG tag do not affect the ability of the E1A peptide to disrupt the CtBP1‐E1A interaction.

### 3.3 CPP‐E1A‐WT peptides can enter cells and interact with CtBP1

**Figure 2:**
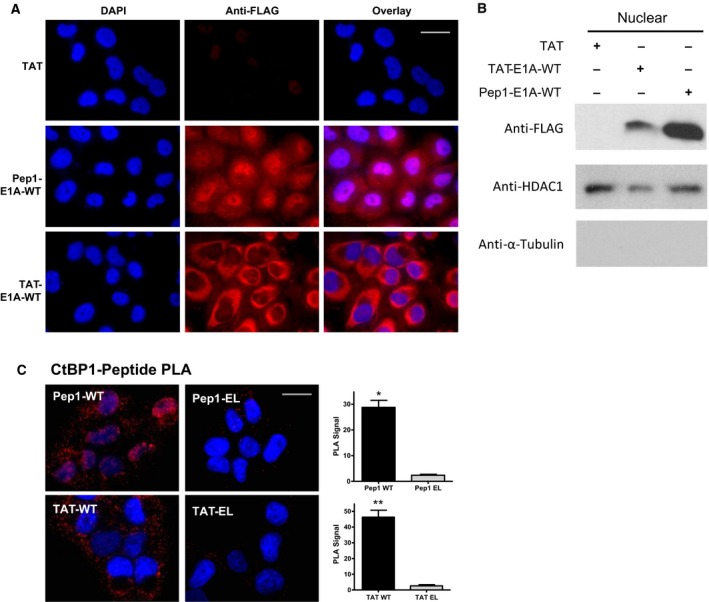
The internalization of the CPP‐fused E1A peptide into cells. (A) Peptide internalization was monitored by immunofluorescence staining with an anti‐FLAG antibody and Alexa Fluor 555 secondary antibody after a 30‐min treatment with 20 μm TAT, TAT‐E1A‐WT, or Pep1‐E1A‐WT. DAPI staining was used to visualize the nucleus. Each figure is the representative image from one of two biological experiments. Scale bar represents 20 μm. (B) Western blot analysis of the nuclear fraction of H1299 cells was used to confirm localization to the nucleus. Anti‐HDAC1 and α‐tubulin antibodies were used as nuclear fractionation and loading controls. This image is a representation of two biological replicates. (C) A PLA was used to observe the CtBP1–peptide interaction in H1299 cells. After a 1‐hour treatment with 10 μm peptide, the protein–peptide interaction was monitored using an anti‐CtBP1 and anti‐FLAG antibody. The quantification of the PLA signal is illustrated to the right of each PLA experiment. Each graph represents the average PLA value quantified from three images taken from two biological replicates. Scale bar represents 20 μm. A paired t‐test statistical analysis was used pairing the WT peptide to the EL peptide at identical concentrations; *P <* *0.05, **P <* *0.01, and ***P <* *0.001.

To evaluate the ability of the TAT and Pep1 peptide sequences to deliver the E1A peptide into cells, immunofluorescence (IF) using an anti‐FLAG antibody was used to monitor peptide internalization in two human cancer cell lines, the p53‐deficient H1299 non‐small cell lung cancer cells (Fig. 2) and the p53 WT A375 melanoma cells (Fig. S1A). Each of the CPP‐fused peptides showed a significant increase in fluorescence compared to cells treated with TAT alone after 30 min (Fig. 2A). In particular, the Pep1‐fused peptide displayed a significant amount of localization to the nucleus, while the TAT‐fused peptide had a much lower amount in the nucleus. To confirm the nuclear localization of each peptide, we performed subcellular fractionation of the H1299 cells and probed the nuclear fraction with an anti‐FLAG antibody on a western blot. Both TAT and Pep1‐fused peptides were found in the nuclear fraction after a 1‐h treatment, and consistent with the IF data, more Pep1‐fused peptide was detected in the nuclear fraction relative to the TAT‐fused peptide (Fig. 2B). These results show that both CPP‐fused peptides are able to internalize into H1299 cells and reach the nucleus. Similarly, we found that both TAT‐ and Pep1‐fused peptides can enter the A375 cells, with Pep1‐E1A exhibiting a more predominant nuclear localization pattern (Fig. S1A).

To determine whether the CPP‐E1A peptides can interact with CtBP1 inside the cell, we monitored the association of these peptides and CtBP1 using a PLA in both H1299 and A375 cells. The WT E1A peptides and CtBP1 protein are within close proximity after a 1‐hour treatment with 10 μm peptides, as revealed by a strong PLA signal (Fig. 2C). In contrast, the mutant E1A‐EL peptide exhibited little PLA signal. These results suggest that only the WT E1A peptide is capable of interacting with CtBP1 inside the cell.

### 3.4 CPP‐E1A‐WT peptides disrupt the CtBP1–ZEB1 interaction and release CtBP1‐mediated repression of target genes

**Figure 3:**
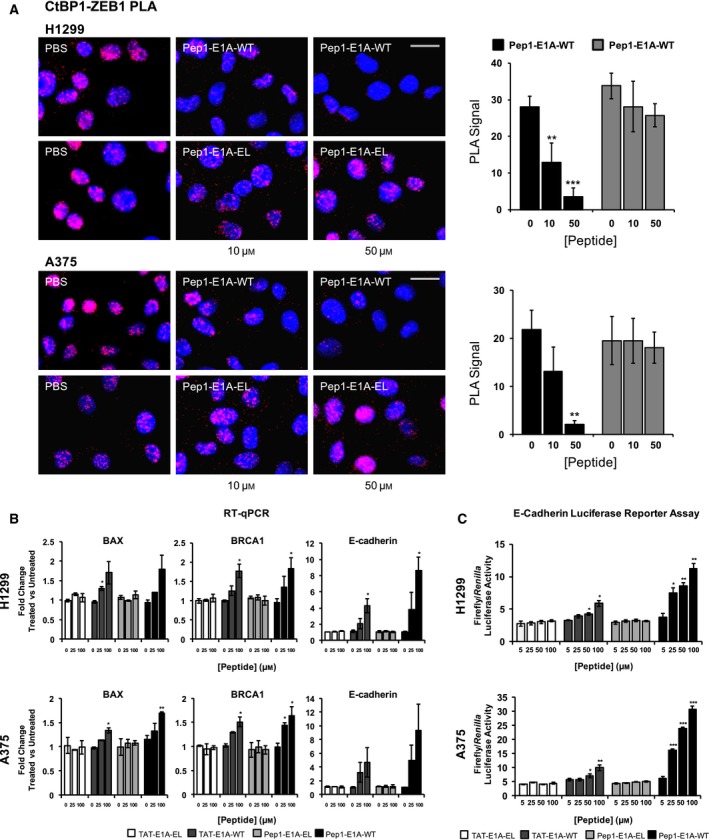
Cell‐penetrating peptide‐fused E1A peptides release CtBP1‐mediated transcriptional repression. (A) A PLA experiment was used to observe the CtBP1–ZEB1 interaction in H1299 and A375 cells. After a 1‐h treatment with 10 and 50 μm peptide, the protein–protein interaction was monitored using an anti‐CtBP1 and anti‐ZEB1 antibody. The quantification of the PLA signal is shown to the right of each PLA experiment. Each graph represents the average PLA value quantified from three images taken from two biological replicates. Scale bar represents 20 μm. The *P*‐value was determined by pairing the WT peptide to the EL peptide of identical concentrations. (B) H1299 and A375 cells were treated with 25 and 100 μm TAT‐E1A‐EL, TAT‐E1A‐WT, Pep1‐E1A‐EL, or Pep1‐E1A‐WT peptides for 5 h, and RT‐qPCR utilizing total mRNA isolated from treated cells was used to detect the mRNA levels of *BAX*,*BRCA1*, and *E‐cadherin*. Each graph represents two biological experiments with three technical replicates, and error bars represent the standard deviation of the averaged value for the biological replicates. Each *P*‐value was calculated by pairing the WT peptides to the TAT peptide of identical concentration. (C) H1299 and A375 cells were transfected with the E‐cadherin promoter‐luciferase construct, and luciferase activity was measured after an 8‐hour treatment with increasing concentrations of either TAT‐E1A or Pep1‐E1A peptides. Each graph represents three experiment, and error bars represent the standard deviation of triplicate samples. *P*‐values were determined comparing the WT peptides to their corresponding EL mutant peptides of identical concentration. *P*‐values represent a paired t‐test statistical analysis in all cases; *P <* *0.05, **P <* *0.01, and ***P <* *0.001.

ZEB1 is a DNA‐binding transcription factor that modulates the expression of key regulatory genes during embryonic development and cell differentiation, including the *E‐cadherin* gene (Eger *et al*., 2005). ZEB1 represses transcription by recruiting CtBP1 through three PXDLS‐like motifs, and the CtBP1–ZEB1 interaction has been shown to downregulate the expression of E‐cadherin (Postigo and Dean, 1999). Furthermore, the expression levels of ZEB1/2 and E‐cadherin are inversely correlative and their expression levels can act as predictors of the epithelial phenotype of lung, melanoma, and other cancer cell lines (Ohira *et al*., 2003; Park *et al*., 2008). To determine whether our CPP‐E1A peptides can disrupt the ability of CtBP1 to interact with its transcription factor partners, we performed a PLA assay monitoring the interaction between CtBP1 and ZEB1, as a representative partner of CtBP1 (Fig. 3A). The strong PLA signal generated by the CtBP1–ZEB1 interaction was not affected by treatment with the mutant Pep1‐E1A‐EL peptide at either 10 or 50 μm for 1 h. In contrast, treatment with the Pep1‐E1A‐WT peptide at 10 and 50 μm resulted in a dose‐dependent decrease in PLA signal after 1 h. These results suggest that the peptides not only can enter cells and bind to CtBP1, but also disrupt the interaction between CtBP1 and its protein partners.

Numerous studies have shown that CtBP1 can suppress the expression of both epithelial and apoptotic genes to promote tumorigenic phenotypes in adult tissues. To assess whether the CPP‐E1A peptides are able to disrupt CtBP1‐mediated transcriptional repression after entering the cell, we performed RT‐qPCR and a luciferase reporter assay on several known CtBP1‐repressed target genes in both H1299 and A375 cells. For the RT‐qPCR analysis, the mRNA level of three CtBP1 target genes, *BAX*,* BRCA1*, and *E‐cadherin*, were monitored after treatment with 0, 25, or 100 μm of TAT alone, TAT‐E1A‐WT, or Pep1‐E1A‐WT (Fig. 3B). For each CtBP1‐target gene, expression levels rose in a dose‐dependent manner for the TAT‐E1A‐WT or the Pep1‐E1A‐WT peptides, whereas the TAT‐E1A‐EL or Pep1‐E1A‐EL mutant peptides showed no significant changes in gene expression (Fig. 3B).

For the luciferase reporter assay, a reporter plasmid carrying the E‐cadherin promoter, a known direct target of CtBP1‐mediated transcription repression, was transfected into H1299 and A375 cells expressing high endogenous levels of CtBP1 (Deng *et al*., 2011, 2013). Luciferase activity increased with increasing concentrations of TAT‐E1A‐WT and Pep1‐E1A‐WT peptides when normalized to a control *Renilla* expression vector, indicating a reversal in the repression of the E‐cadherin promoter (Fig. 3C). However, the mutant peptides TAT‐E1A‐EL and Pep1‐E1A‐EL showed no significant loss of repression of the E‐cadherin promoter. These results strongly suggest that the CPP‐fused E1A peptides are able to enter into cells and disrupt CtBP1‐mediated repression of target genes. These results illustrate the potential of using peptides containing the PXDLS‐binding motif to disrupt CtBP1 transcriptional activity as an anticancer therapeutic.

### 3.5 Constitutive expression of the CPP‐E1A‐WT peptide in melanoma cells inhibits oncogenic phenotypes in culture and limits tumor growth *in vivo*

**Figure 4:**
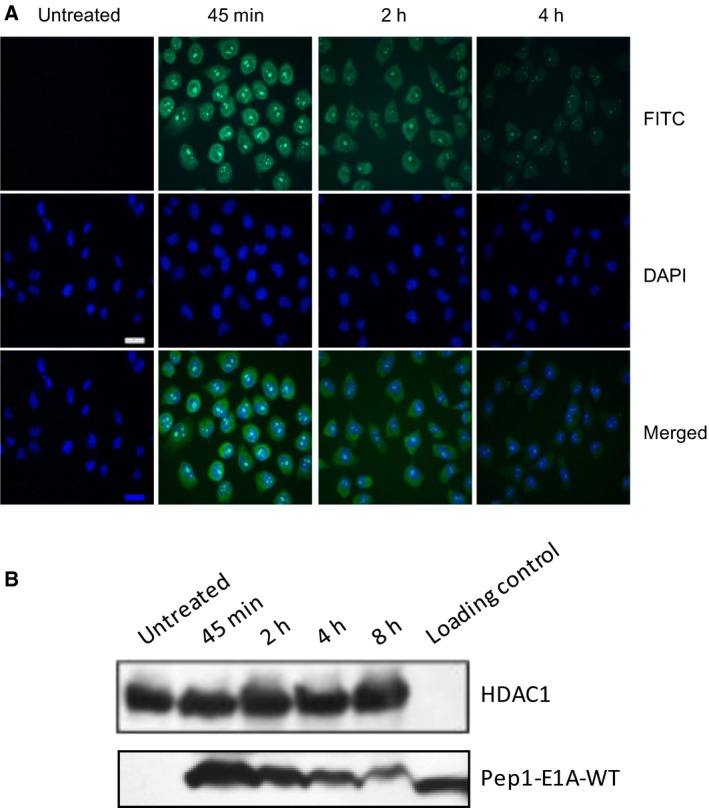
Pep1‐E1A‐WT has a short half‐life *in vitro*. (A) Peptide stability was monitored in H1299 cells after treatment with 10 μm FITC‐labeled Pep1‐E1A‐WT for 45 min, 2 h, and 4 h, washed six times with PBS, and visualized by fluorescence microscopy. DAPI staining was used to visualize the nucleus. Each image is a representation of at least two images taken from three experimental replicates. Scale bar represents 25 μm. (B) Western blot analysis of the whole‐cell lysate of H1299 cells after treatment with 10 μm of Pep1‐E1A‐WT for 45 min, 2 h, 4 h, and 8 h. Pep1‐E1A‐WT peptide was detected using an anti‐Flag antibody. This image is a representation of two biological replicates.

An increase in CtBP1 expression levels promotes cellular proliferation in several cancers (Bergman and Blaydes, 2006). As the CPP‐fused peptides containing the WT E1A sequence were able to release CtBP1‐mediated gene repression, as determined by RT‐qPCR and a luciferase reporter assay, we hypothesized that these peptides would also reverse CtBP1‐mediated oncogenic phenotypes. However, therapeutic peptides often suffer from a high degree of proteolytic degradation *in vivo* (Trehin *et al*., 2004)*,* resulting in shortened half‐lives when compared to small molecule therapeutics. This is consistent with our preliminary findings that the peptide level in cells is significantly reduced within 2–8 h of treatment by western blot analysis and fluorescence microscopy of FITC‐labeled CPP‐E1A‐WT peptide (Fig. 4). Additionally, short time points (5–8 h) were required to detect changes in CtBP1‐mediated repression in both the RT‐qPCR and luciferase reporter assays. Inhibition of CtBP1‐mediated repression was undetectable when examined at 10 h by RT‐qPCR and 16 h in the luciferase reporter assay (data not shown).

**Figure 5:**
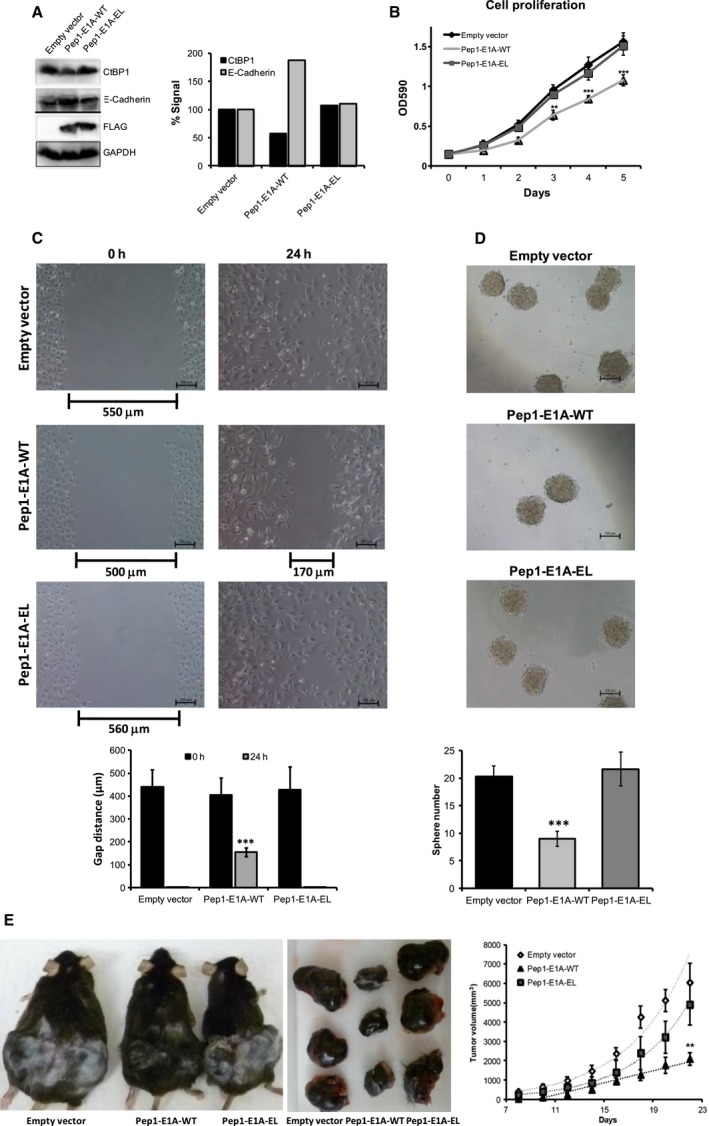
The Pep1‐E1A‐WT peptide reverses oncogenic phenotypes. (A) E‐cadherin, CtBP1, and peptide expression levels were examined by western blot analysis in A375 cells stably expressing the Pep1‐E1A‐WT and EL fusion peptides. Graph to the right of the bands indicate the percentage of signal compared to the empty vector (set to 100%) after being normalized to GAPDH. (B) The proliferation of the A375 cells overexpressing the Pep1‐E1A‐WT peptides was monitored using crystal violet staining for 5 days. *P*‐values were determined comparing the WT peptides to the EL mutant peptide at identical time points. (C) A 24‐h migration assay was performed on the A375 cells stably transfected with the empty vector, Pep1‐E1A‐WT, or Pep1‐E1A EL. Quantification from two biological replicates of the gap distance is located below the representative images. The *P*‐value was determined comparing the gap distance for the WT peptide to the EL peptide at 24 h. (D) A 24‐hour sphere formation assay was carried out with the stable A375 cell lines. The numbers of spheres from two biological replicate experiments are illustrated below the representative images from each cell line. The *P*‐value was determined comparing the sphere number for the WT peptide and the EL peptide. (E) C57BL/6 mice were subcutaneously injected with 1 × 10^7^ of B16‐F0 cells stably transfected with the vector control, Pep1‐E1A‐WT, or Pep1‐E1A‐EL, on both left and right back flanks. Tumor growth was observed over time and measured by caliper measurements for 21 days before mice were sacrificed. The *P*‐value was determined comparing the tumor size for the WT peptide and the EL peptide on identical days. *P*‐values represent a paired t‐test statistical analysis in all cases; *P <* *0.05, **P <* *0.01, and ***P <* *0.001.

To circumvent the relatively short half‐life of the CPP‐E1A peptides and to evaluate their potential effect on oncogenic phenotypes, we decided to constitutively express the CPP‐E1A peptides in cancer cell lines. We chose to focus on the Pep1‐fused peptides, as the Pep1‐E1A‐WT peptide was more efficient than the TAT fusion at entering the nucleus and reversing CtBP1‐mediated transcription in the RT‐qPCR and luciferase assays. The human melanoma cell line A375 was chosen because of its highly aggressive oncogenic phenotypes that are dependent upon CtBP1 overexpression (Deng *et al*., 2013). Stably transfected A375 lines expressing comparable level of the Pep1‐E1A‐WT and Pep1‐E1A‐EL peptides were generated (Fig. 5A). We observed an increase in E‐cadherin expression levels in cells expressing the WT peptide as compared to the EL mutant, by western blot analysis (Fig. 5A). Interestingly, we saw a decrease in CtBP1 protein levels in cells expressing the WT peptides relative to the EL mutant (Fig. 5A), suggesting that CtBP1 may be prone to proteolysis when not bound to its transcription factor partners. Importantly, we found A375 cells expressing the Pep1‐E1A‐WT peptide displayed a significant decrease in cell proliferation, cell migration, and sphere‐forming ability (Fig. 5B–D).

Previously, CtBP1 overexpression has been demonstrated to be important for suppressing tumor suppressor genes and promoting melanoma development (Deng *et al*., 2013). To determine whether these peptides can limit tumor growth induced by CtBP1 *in vivo*, we performed a tumor xenograft growth assay using stably transfected mouse melanoma B16‐F0 cells expressing vector control, Pep1‐E1A‐WT, or Pep1‐E1A‐EL. *In vitro* analyses revealed that the Pep1‐E1A‐WT expressing B16‐F0 cells also exhibited significantly reduced proliferation, migration, and sphere‐forming abilities relative to the vector control and the Pep1‐E1A‐EL mutant (Fig. S2). These cell lines were subcutaneously injected into C57BL/6 mice on the back flanks. B16‐F0 injected mice form palpable tumors within 10 days, and the tumors were monitored by caliper measurement until 21 days postinjection. Mice injected with B16‐F0‐Pep1‐E1A‐WT cells exhibited a significant reduction in tumor growth in comparison with those injected with B16‐F0‐vector control or B16‐F0‐Pep1‐E1A‐EL (Fig. 5E). Additionally, our preliminary observation indicates that mice injected with B16‐F0‐Pep1‐E1A‐WT cells had an overall better survival rate compared to mice injected with B16‐F0‐vector control or ‐Pep1‐E1A‐EL cells (data not shown). These data demonstrate that disruption of the CtBP1–protein partner interaction potently inhibits the ability of CtBP1 to promote tumor growth. Together, these results strongly indicate that these peptides may be a useful strategy to reverse tumorigenesis in melanoma, lung, and possibly other cancers where the CtBP1 corepressor is overexpressed.

## 4 Discussion

The interaction between CtBP1 and transcription factors is essential for the suppression of many genes involved in cellular activities important for both development and oncogenesis. Although CtBP expression is low in most adult tissues, it is often overexpressed in multiple types of cancer where it confers developmental phenotypes out‐of‐context (Byun and Gardner, 2013). In addition, the elevated NADH levels associated with hypoxia in solid tumors can further increase CtBP activity (Zhang *et al*., 2006). Importantly, disrupting CtBP function can promote apoptosis in a p53‐independent manner. Therefore, the disruption of CtBP‐mediated gene repression could be an effective approach to target cancer cells, especially those with mutant p53, while sparing normal tissues. In particular, the ability of the E1A peptide to competitively inhibit CtBP–protein partner interactions makes this peptide a potentially attractive foundation for the development of peptide‐based antineoplastic therapeutics.

Compared to small molecules, peptide inhibitors typically have high specificity and low toxicity. However, development of peptide therapeutics often faces two hurdles, cell impermeability and degradation/proteolysis. Fusion of the E1A peptide at the N terminus with CPP facilitates cell entry and CtBP1 inhibition in H1299 lung carcinoma and A375 melanoma cells, overcoming the first hurdle. As shown in Fig. 4, our peptides exhibit limited stability *in vitro*. H1299 and A375 cells have a population doubling time of approximately 30 and 32 h (Holbrook *et al*., 2009; Lotan, 1979), which is much longer than the peptide half‐life, potentially making any changes in cell proliferation difficult to detect. Therefore, strategies to extend the peptide half‐life should be an important consideration during future peptide optimization. Possible approaches include utilizing structural modifications, such as incorporating non‐natural amino acids, N‐ and C‐terminal modifications, pseudo‐peptide bonds, and peptide cyclization (Di, 2015). Furthermore, some of these modifications will not only improve peptide stability, but may also improve other peptide pharmacological properties; for example*,* cyclization can increase both stability and permeability (Ovadia *et al*., 2010).

Interestingly, we noticed a decrease in CtBP1 protein levels in the A375 cells that express the E1A‐WT peptide (Fig. 5A), suggesting that CtBP1 is unstable when it is not bound to its protein partners. This is supported by the work of Choi *et al*. (2010) who found that the binding of Bcl3 to CtBP1 through the conserved PXDLS motif blocks ubiquitination of CtBP1, leading to CtBP1 stabilization. It is possible that our smaller E1A peptide, while competent in competing for CtBP‐binding against its endogenous protein partners, is insufficient to block CtBP1 phosphorylation at the S422 residue, which ultimately leads to its ubiquitination and degradation. Therefore, our results suggest that these peptides may work by both inhibiting the formation of the corepressor complex and reducing CtBP1 protein levels by facilitating its degradation.

In summary, we have developed an E1A peptide capable of entering cells and disrupting CtBP1‐mediated transcriptional repression. These peptides clearly release CtBP1‐mediated transcriptional repression judging by both expression level of endogenous target genes and a luciferase assay using a CtBP1‐specific promoter. Furthermore, the constitutive expression of these peptides can reverse CtBP1‐mediated oncogenic phenotypes in a melanoma model in both cell culture and mice. Importantly, the results highlight the exciting possibility of peptide therapeutics for treating cancers with CtBP overexpression or hyperactivity. Future efforts should be focused on creating more stable peptides with increased resistance to proteolytic degradation and improving peptide potency by identifying peptides with lower IC_50_ values.

## Author contributions

MAB, MH, and RZ conceived and designed experiments; MAB, CZ, LZ, HL, and XL conducted experiments; MAB, DAN, MH, and RZ secured funding for the project; MAB, MH, and RZ wrote the manuscript.

## Supporting information


**Fig. S1.** Peptides internalize into A375 cells and binds CtBP1.
**Fig. S2.** The Pep1‐E1A‐WT peptide reverses oncogenic phenotypes in B16‐F0 cells.
