# Supplementary material for: CPP‐E1A fusion peptides inhibit CtBP‐mediated transcriptional repression
Source: Mol Oncol. 2018 Jun 23;12(8):1358–73. doi: 10.1002/1878-0261.12330 (PMC6068344; doi:10.1002/1878-0261.12330)
Supplement: Supplementary file 1 — Fig. S1. Peptides internalize into A375 cells and binds CtBP1. Fig. S2. The Pep1‐E1A‐WT peptide reverses oncogenic phenotypes in B16‐F0 cells. [file MOL2-12-1358-s001.pdf]

Supplemental Figure 1

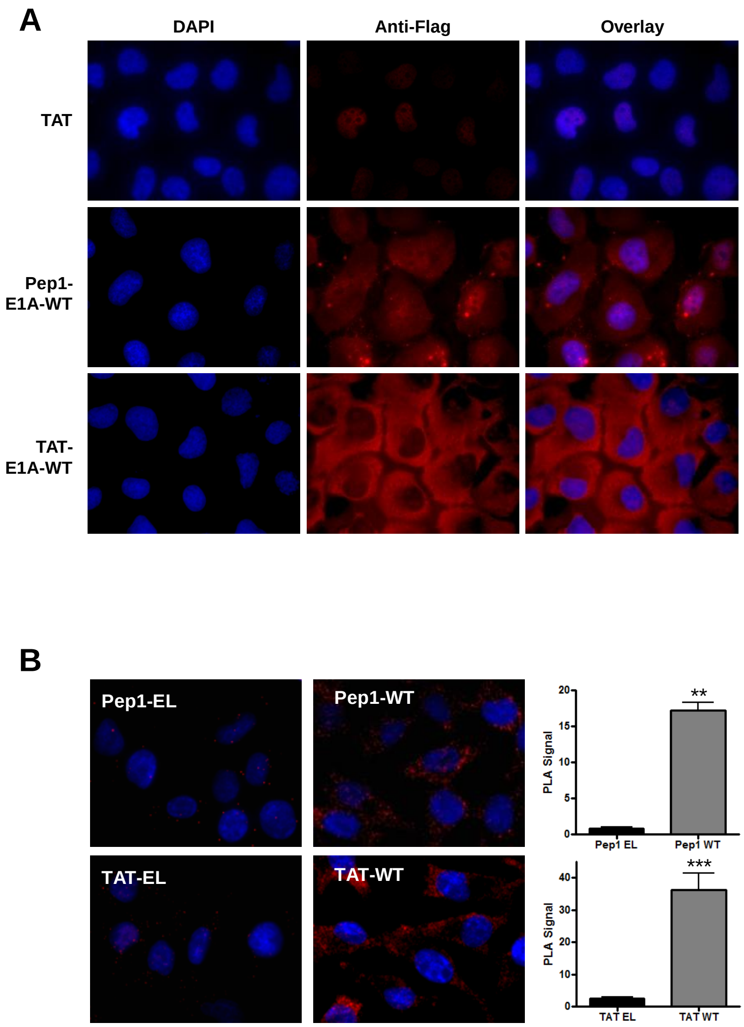

Supplemental Figure 2

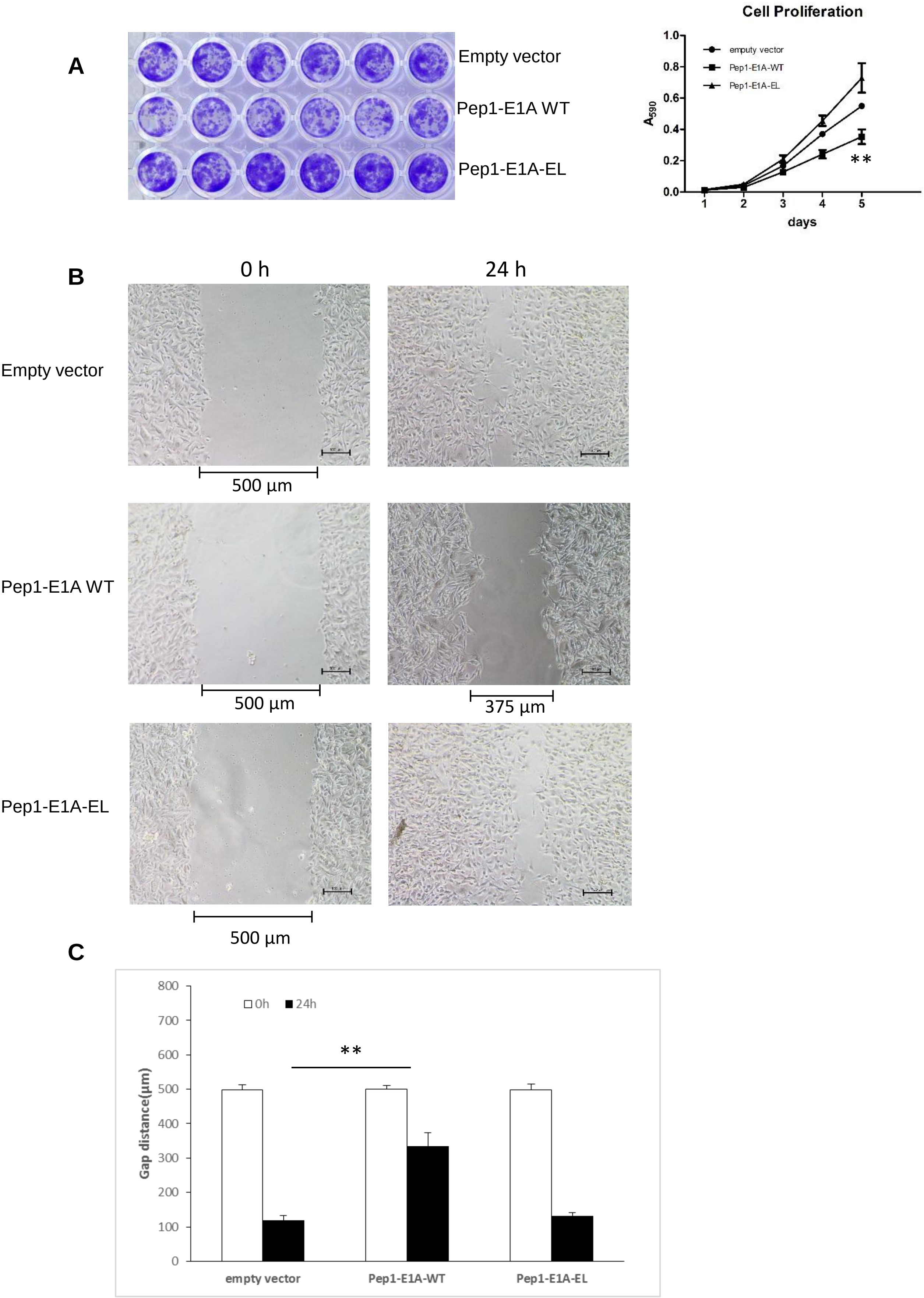

Supplemental Figure 1 **(A)** Peptide internalization into A375 cells was monitored by immunofluorescence staining with an anti-FLAG antibody and Alexa Fluor 555 secondary antibody as described in Figure 2. **(B)** A PLA was used to observe the CtBP1-peptide interaction in A375 cells as also described in Figure 2.

Supplemental Figure 2 B16-F0 cells stably transfected with the empty vector, Pep1-E1A-WT and Pep1-E1A-EL were analyzed for proliferation **(A)**, migration **(B)**, and sphere-forming abilities **(C)** as described in Figure 5.
